# Inactivation of the conserved protease LonA increases production of xylanase and amylase in *Bacillus subtilis*

**DOI:** 10.1186/s12934-024-02616-6

**Published:** 2024-12-19

**Authors:** Biwen Wang, Mariah B. M. J. Kes, Anna C. H. van den Berg van Saparoea, Gaurav Dugar, Joen Luirink, Leendert W. Hamoen

**Affiliations:** 1 Swammerdam Institute for Life Sciences, University of Amsterdam, Science Park 904, C3.108, Amsterdam, 1098 XH The Netherlands; 2 Molecular Microbiology, AIMMS and A-LIFE, Vrije Universiteit Amsterdam, Amsterdam, 1081 HZ The Netherlands

**Keywords:** *Bacillus*, Xylanase, Amylase, Secretion, Transcriptome profiling, Ribosome profiling, CtsR, LonA

## Abstract

**Background:**

*Bacillus subtilis* is widely used for industrial enzyme production due to its capacity to efficiently secrete proteins. However, secretion efficiency of enzymes varies widely, and optimizing secretion is crucial to make production commercially viable. Previously, we have shown that overexpression of the xylanase XynA lowers expression of Clp protein chaperones, and that inactivation of CtsR, which regulates and represses *clp* transcription, increases the production of XynA. In the current study, we examined whether the same is the case for overexpression of the α-amylase AmyM from *Geobacillus stearothermophilus* by *B. subtilis*, and why XynA shows a different timing of secretion compared to AmyM.

**Results:**

Transcriptome analyses revealed that *B. subtilis* cells overexpressing AmyM exhibited a distinct profile compared to XynA overexpressing cells, however there were also similarities and in both cases expression of CtsR controlled genes was downregulated. In contrast to XynA, inactivation of CtsR did not improve AmyM production. Upregulation of other protein chaperones, including GroEL/ES and DnaJ/K, by inactivating their transcriptional repressor HrcA, had almost no effect on XynA yields and in fact considerably lowered that of AmyM. Despite using the same promoter, the production of XynA peaks well before AmyM reaches its optimal secretion rate. Transcriptome and ribosome profiling indicated that this is neither related to transcription nor to translation regulation. We show that the reduced secretion in the stationary phase is partially due to the activity of secreted proteases, but also due to the activity of the intracellular protease LonA. The absence of this protein resulted in a 140% and 20% increased production for XynA and AmyM, respectively.

**Conclusion:**

The combination of transcriptome and ribosome profiling offered important information to determine at which cellular level production bottlenecks occurred. This helped us to identify LonA protease as an important factor influencing enzyme production yields in *B. subtilis*.

**Supplementary Information:**

The online version contains supplementary material available at 10.1186/s12934-024-02616-6.

## Introduction

*B. subtilis* is a gram-positive nonpathogenic and generally regarded as safe (GRAS) bacterium [1], and is widely used as cell factory for the industrial-scale production of enzymes for the detergents, food, beverages, paper and pharmaceutical industries [2–5]. To date, approximately 60% (in weight) of the commercially available enzymes are produced by *Bacillus* species [2]. However, the range of proteins that is efficiently secreted by *Bacilli* is limited. Attempts to improve secretion of other enzymes typically involve testing different promoters and secretion signal sequences, eliminating extracellular proteases and optimizing fermentation conditions [6–8]. Despite many years of research, it is still not clear why some enzymes are secreted at higher levels than others.

In a previous study we used transcriptome profiling to identify cellular stresses when *B. subtilis* cells overexpress the endogenous endo-1,4-β-xylanase XynA, an enzyme used in the paper and textile industries [9], and found that XynA overexpression leads to the down-regulation of the Clp chaperone genes *clpC*/*E*/*X*, and their related protease gene *clpP*. Upregulation of these genes, by inactivating their repressor CtsR, improved XynA production by 20–30% [10]. In the current study, we have investigated whether this approach can also increase the production of other enzymes, such as the industrial relevant maltogenic α-amylase AmyM from *Geobacillus stearothermophilus.* This protein, which is used in the food, brewing, and textile industry [11, 12], has a molecular weight of 75.4 kDa and is much larger than XynA (20.4 kDa). We first performed a transcriptome analysis of AmyM overexpressing *B. subtilis* cells and found that the transcriptome profile differed substantially from XynA overexpressing cells, although the *clp* genes were downregulated in both cases. However, inactivation of CtsR did not improve AmyM production. Inducing the expression of other protein quality control genes, including *dnaK*, *dnaJ*, and *groEL*/*ES*, by deleting the repressor HrcA [13], neither improved XynA nor AmyM production.

During our expression studies we noticed that the secretion of XynA peaked well before that of AmyM. However, the transcriptome data showed that there is abundant *xynA* mRNA present when AmyM secretion is optimal. To examine whether the *xynA* mRNA is expressed, we performed a ribosome profiling experiment [14–16]. This showed that *xynA* mRNA is normally translated, suggesting that XynA is degraded before it can be secreted. Interestingly, when we deleted the common class III heat-shock and ATP-dependent protease LonA [17], the production of XynA continued and increased 2.4-fold. Inactivation of LonA increased AmyM production by up to 20%. LonA is almost universally conserved, suggesting that the inactivation of this gene will improve protein secretion in many production organisms.

## Results

### Transcriptome profiles of XynA and AmyM overexpressing cells

**Fig. 1:**
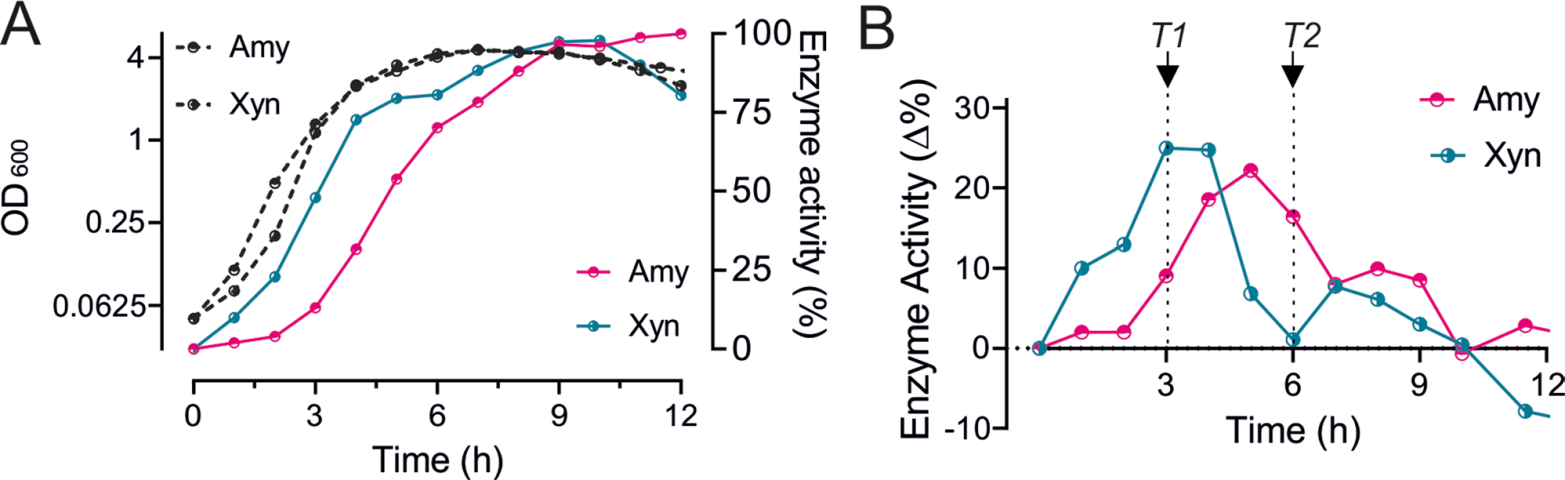
Comparison of amylase (Amy) and xylanase (Xyn) overproduction in *B. subtilis* BWB09. (**A**) Growth curves and enzyme activities of AmyM (Amy) and XynA (Xyn) overexpressing strain BWB09/pCS74 and BWB09/pCS58, respectively. (**B**) Change in enzyme activities (Δ mU/mL) over 1 h time intervals. Arrows indicate the sampling timepoints T1 and T2 for RNA-seq

Previously, we have shown that the overproduction of XynA downregulates the expression of *Clp* chaperone genes, and when this was prevented by deleting the CtsR repressor, production of XynA increased by 25%. CtsR is under proteolytic control of ClpCP [18, 19], and it was argued that overproduction of XynA competes with this proteolytic control, resulting in more active CtsR and subsequent downregulation of *clp* genes [10]. Therefore, we were curious whether this downregulation also occurs when other enzymes are overproduced, such as AmyM. We began by determining whether the *clp* genes are also downregulated during AmyM overexpression using transcriptome profiling. To find the relevant time points for mRNA isolation, we first determined the secretion profiles of both XynA and AmyM. Overexpression of these proteins was achieved using the constitutive *amyQ* promoter and a high copy plasmid [10]. The *B. subtilis* strain used as host, BWB09, was unable to sporulate due to the inactivation of the crucial sporulation regulator gene *spoIIE*. A strain containing the plasmid that lacked these genes was used as negative control for the enzyme assays, and as reference for the differential expression calculations. Strains were grown in nutrient-rich LB medium at 37 °C with 50 µg/mL kanamycin to maintain the plasmids. The growth of the different cultures and the xylanase and amylase enzyme activities in the medium are shown in Fig. 1. XynA secretion begins already during the logarithmic growth phase but slows down when the culture enters the stationary phase. AmyM production commences later when the stationary phase begins. Therefore, we sampled cells for mRNA isolation around 3 h and 6 h growth (Fig. 1). A principal component analysis (PCA) indicated good biological replicates (Figure S1).

Tables 1 and 2 lists the 12 most up- and downregulated genetic loci for AmyM and XynA overproduction at 3 h and 6 h, respectively. Of note, the 3 h XynA transcriptome data originated from our previous study [10]. Tables 1 and 2 shows that there is a clear difference in gene expression response between cells expressing either AmyM or XynA. For example, amylase overexpression induces the expression of the membrane-anchored protein quality control proteases HtrAB, as has been shown before [20], but this was not observed for XynA overexpression. There are also several genes that show a comparable change in expression upon overexpression of AmyM and XynA, including a number of metabolic genes, such as those involved in pyrimidine biosynthesis and various sugar utilization genes. Additionally, there is common downregulation of a few prophage genes and the AAA unfoldase encoding *clpE* gene (Tables 1 and 2).

**Table 1:** 12 most significantly up-/downregulated genetic loci upon either AmyM or XynA overexpression (p value < 0.05), after 3 h growth. Genes of the same operon that follow similar regulation are clustered

log_2_FC Amy3h	log_2_FC Xyn3h	gene(s)	regulon	function
**11.05**	n.a.	*amyM*		exogenous amylase
**3.80**	0.00	*htrB*	CssR	protein quality control
**3.60**	1.10	*phrF*	ComA	control of ComA activity
**3.60**	0.00	*htrA*	CssR	protein quality control
**3.20**	0.30	*phrC*	ComA	control of ComA activity
**2.4/1.2**	1.6/0.7	*pyrAA*, *AB*, *C*,*D*, *E*,*F*, *K*,*P*	PyrR	pyrimidine biosynthesis
**2.2/1.1**	1.3/0.1	*tuaA/2*,*C*, *D*	PhoP	teichuronic acid biosynthesis
**2.16**	0.10	*phrA*	ComA	control of sporulation initiation
**1.87**	0.50	*pstS*	PhoP	high-affinity phosphate uptake
**1.8/1.1**	1.4/-1.0	*uxaA*, *C*,*yjmF*	ExuR	hexuronate utilization
**1.79**	0.50	*dppD*	CodY	uptake of dipeptides
**1.65**	-0.51	*phrE*	ComA	control of sporulation initiation
0.03	**11.43**	*xynA*		endogenous xylanase, xylan degradation
1.9/-0.3	**2.7/0.3**	*yodS*, *R*,*Q*, *P*,*kamA*	SigE	unknown, similar to butyrate-acetoacetate CoA-transferase
-0.1/-0.8	**2.6/0.7**	*glnQ*, *H*,*P*	SigE	glutamine uptake
0.30	**2.5/1.3**	*yxeK*, *N*,*M*, *O*,*Q*, *snaB*, *sndB*	CymR	utilization and detoxification S-(2-succino) cysteine
0.50	**2.40**	*yvdE*		regulation of starch and maltodextrin utilization
1.5/-1.6	**2.0/0.8**	*snaA*, *tcyJ*, *K*,*L*, *M*,*N*, *cmoO*, *I*,*J*, *ribR*, *sndA*, *ytnM*	CymR	utilization of S-methyl-cysteine
0.8/0.4	**1.9/1.2**	*cysH*, *P*,*C*, *sat*, *ylnD*	CymR	sulfate uptake & reduction
2.4/1.2	**1.6/0.7**	*pyrAA*, *AB*, *C*,*D*, *E*,*F*, *K*,*P*	PyrR	pyrimidine biosynthesis
0.16	**1.57**	*tcyP*	CymR	cystine (cysteine dimer) uptake
-1.17	**1.46**	*spoVID*	SigE	spore coat assembly
1.8/-0.1	**1.3/1.2**	*dppB*, *C*,*D*	CodY	dipeptide transporter subunit
0.4/-1.7	**1.3/0.4**	*nupN*, *O*,*P*, *Q*	CodY	dipeptide transporter subunit
**-3.45**	-0.34	*yoqH*		unknown, SP-beta prophage
**-3.10**	0.50	*spsA*	GerE	spore coat polysaccharide synthesis
**-2.96**	-0.47	*ynzG*		unknown
**-2.90**	-2.00	*clpE*	CtsR	AAA unfoldase
**-2.6/-1.7**	-2.2/-0.8	*melR*, *E*,*D*, *C*	MelR	regulation of melibiose utilization
**-2.40**	-0.20	*sirA*	Spo0A	control of chromosome copy number
**-2.36**	0.47	*yqbB*		unknown, skin element
**-2.10**	-1.60	*iseA*	WalR	protection against cell envelope stress
**-2.07**	0.23	*yjcL*		unknown, skin element
**-1.90**	0.90	*narG*	Fnr	nitrate respiration, nitrogen assimilation
**-1.9/-1.1**	-1.5/-0.9	*araA*, *B*,*D*, *L*,*M*, *N*,*P*	AraR	arabinose utilization
**-1.70**	0.40	*nupQ*	CodY	guanosine uptake
-0.24	**-2.87**	*yosA*		unknown, SP-beta prophage
-2.6/-1.7	**-2.2/-0.8**	*melR*, *E*,*D*, *C*	MelR	regulation of melibiose utilization
-2.90	**-2.00**	*clpE*	CtsR	AAA unfoldase
-2.10	**-1.60**	*iseA*	WalR	protection against cell envelope stress
-1.4/-1.1	-1.6/-1.3	*yyzE*, *bglA*		unknown, beta-glucoside utilization
-0.9/-0.6	**-1.6/-1.4**	*rbsA*, *B*,*C*, *D*,*K*, *R*	CcpA	ribose uptake
-1.4/1.1	**-1.6/1.3**	*yyzE*, *bglA*		beta-glucoside utilization
-1.6/-1.2	**-1.6/-1.2**	*licA*, *B*,*C*, *H*	LicR	lichenan uptake and phosphorylation
-1.9/-1.1	**-1.5/-0.9**	*araA*, *B*,*D*, *L*,*M*, *N*,*abfA*	AraR	arabinose utilization
-1.42	**-1.48**	*glpD*	GlpP	glycerol-3-phosphate dehydrogenase
-0.30	**-1.32**	*ywsB*	SigB	general stress protein
-1.4/-1.1	**-1.3/-1.1**	*khtT*, *U*,*S*		potassium ion efflux

**Table 2:** 12 most significantly up/down regulated genetic loci upon either AmyM or XynA overexpression after 6 h growth (p value < 0.05). Genes of the same operon that follow similar regulation are clustered

log_2_FC Amy6h	log_2_FC Xyn6h	gene(s)	regulon	function
**15.04**	**n.a.**	*amyM*		exogenous amylase in the plasmid
**5.30**	0.50	*htrA*	CssR	protein quality control
**5.20**	0.50	*htrB*	CssR	protein quality control
**4.39**	-0.09	*ykoJ*		unknown
**2.3/1.9**	2.00	*cssR/S*	CssR	protein secretion stress regulator
**1.90**	-0.20	*yirB*	CssR	control of proteolysis
**1.9/1.4**	0.3/0	*uxaC*, *uxuA*, *yjmB*, *C*	ExuR	hexuronate utilization
**1.4/1.2**	0.7/0.4	*ykuN*, *O*,*P*	Kre	electron transfer
**1.38**	0.13	*yusZ*		unknown, putative short-chain acyl dehydrogenase
**1.40**	1.60	*sr1*	CcpN	control of arginine metabolism, glycolysis & sporulation
**1.10**	0.50	*yxeB*	Fur	siderophore uptake
**1.10**	-0.50	*lctP*	Rex	lactate excretion
0.35	**11.74**	*xynA*		endogenous xylanase, xylan degradation
1.76	**4.26**	*yqaF*	SknR	similar to transcription regulator, skin element
1.40	**1.60**	*sr1*	CcpN	control of arginine metabolism, glycolysis & sporulation
0.41	**1.44**	*ydjB*		unknown, Prophage 3
0.39	**1.40**	*yuiA*		unknown
1.06	**1.4/0.9**	*gapB*, *speD*	CcpN	anabolic enzyme in gluconeogenesis
0.5/0.2	**1.3/0.7**	*ribD*, *E*,*A*, *H*	RibR	riboflavin biosynthesis
0.4/0.3	**1.2/0.8**	*leuB*, *C*,*D; ilvB*, *H*,*C*	CcpA	biosynthesis of leucine and branched-chain amino acids
0.5/0.4	**1.1/0.5**	*natA*, *B*	NatR	sodium export
1.03	**1.04**	*pckA*	CcpN	synthesis of phosphoenolpyruvate
-0.43	**1.01**	*frlR*		regulation of utilization of sugar amines
0.1/0.0	**1.0/0.7**	*ykfA*, *B*,*C*, *D*	CodY	cell wall metabolism and immunity to bacteriotoxins
**-3.80**	-3.80	*clpE*	CtsR	AAA unfoldase
**-1.70**	-2.70	*iolT*	IolR	inositol utilization
**-1.47**	-2.14	*yodA*		unknown, similar to malonate semialdehyde decarboxylase
**-1.45**	-0.60	*spoIIB*	SigV	spore morphogenesis
**-1.43**	-0.56	*yomU*		unknown, SP-beta prophage
**-1.40**	-0.40	*rsbRD*	SigB	negative regulator of SigB
**-1.37**	-1.13	*ydjJ*		unknown
**-1.30**	-1.40	*nasD*	Fur	nitrite utilization
**-1.20**	-1.00	*iseA*	WalR	protection against cell envelope stress
**-1.9/-1.2**	-3.4/-1.3	*sboA*, *X*, *albA*, *B*,*C*, *D*,*E*, *F*,*G*	AbrB	antilisterial subtilosin production
**-1.2/-0.9**	-3.7/-3	*melA*, *D*,*E*, *R*	MelR	melibiose utilization
**-1.10**	-0.80	*comGA*	ComK	genetic competence
-3.80	**-3.80**	*clpE*	CtsR	AAA unfoldase
-1.2/-0.9	**-3.7/-3**	*melA*, *D*,*E*, *R*	MelR	melibiose utilization
-1.9/-1.2	**-3.4/-1.3**	*sboA*, *X*, *albA*, *B*,*C*, *D*,*E*, *F*,*G*	AbrB	antilisterial subtilosin production
-1.9/-1.0	**-2.9/-1.8**	*iolA*, *B*,*C*, *D*,*E*, *F*,*G*, *H*,*I*	IolR	myo-inositol catabolism
-1.70	**-2.70**	*iolT*	IolR	inositol utilization
-0.41	**-2.62**	*yebD*		unknown
-1.06	**-2.52**	*fnr*	ResD	transcriptional regulator of anaerobic genes
-0.89	**-2.26**	*yojB*		unknown
0.05	**-2.20**	*trnB-Arg*		transfer RNA-Arg
-0.75	**-2.2/-2.1**	*ycnI*, *J*,*K*	YcnK	regulation of copper uptake
-0.75	**-2.20**	*ctaA*	ResD	heme biosynthesis
-0.1/0	**-2.2/-1.9**	*qoxA*, *B*,*C*, *D*	CitB	cytochrome aa3 quinol oxidase

### Comparing transcriptome profiles using regulon information

**Fig. 2:**
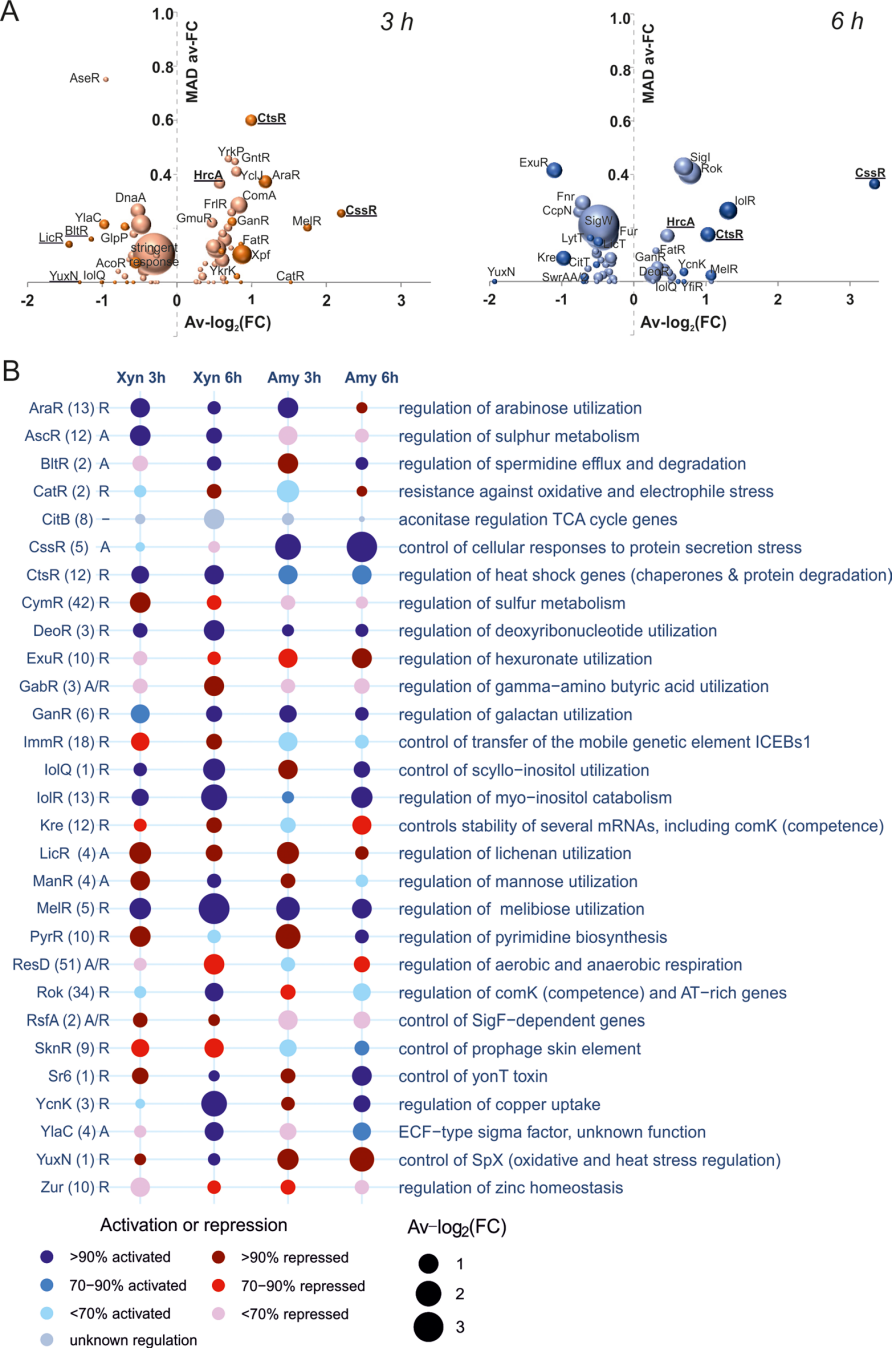
Comparison of regulon changes between XynA and AmyM overexpressing cells. (**A**) GINtool bubble plots for AmyM overexpressing cells showing the average fold change of regulons in log_2_ scale (Av-log_2_(FC)) plotted against the spread in fold-change of regulon genes, expressed as mean absolute deviation (MAD av-FC). Bubble sizes reflect the number of genes of a regulon. Colour intensities relate to average p values, which have been classified into five categories: <0.0625, < 0.125, < 0.25, < 0.5. For clarity, regulons with average p values > 0.25 are not shown. Positive and negative average fold change values relate to activated and repressed regulators, respectively. (**B**) Bubble-matrix chart showing the 10 most altered regulons for XynA and AmyM overexpressing cells at 3 h and 6 h. Total number of genes of a regulon is indicated between bracket. Transcriptional activators are indicated by “A”, repressors by “R”, regulators with both activities by “A/R” and regulators with unknown activity by “-“. Colour intensities reflect the percentage of regulon genes that correspond to the most likely activity of the regulator, bubble sizes correspond to the average fold chance of regulons

**Fig. 3:**
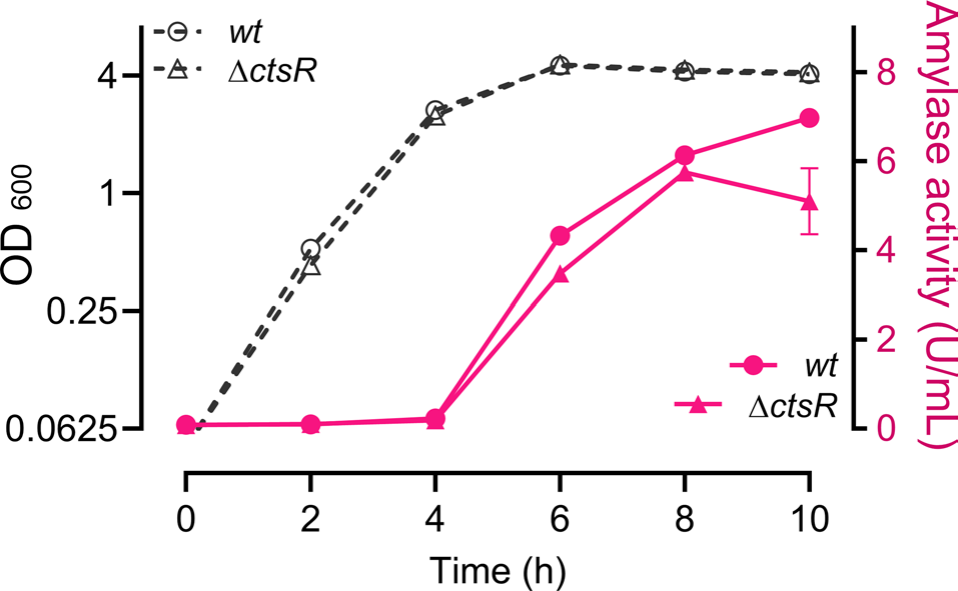
Effect of *ctsR* inactivation on AmyM production. Growth (OD_600_) and amylase activity in the medium of strains BWB09/pCS74 (*wt*) and SGB03/pCS74 (∆*ctsR*) overexpressing AmyM. Data shown as mean ± standard deviation of two biological replicates

Expression of *clpE*, like the other *clp* genes, is controlled by the transcriptional repressor CtsR [21, 22]. However, from Tables 1 and 2 it is unclear whether the expression of other *clp* genes is affected as well. Therefore, we used a gene set enrichment analysis tool that can use regulon information, GINtool [10, 23], to see how regulons are affected, including the CtsR regulon. As shown in the bubble plot of Fig. 2A, regulation of the CtsR regulon is clearly affected after both 3 h and 6 h overexpression of AmyM. Of note, the positive fold change value indicates that the repressor CtsR is active, because the CtsR regulon genes are downregulated. This distinction is necessary since some regulators can function both as activator and as repressor [23]. To compare the difference in regulon activities between conditions, we plotted the 10 most affected regulons for each condition and time point in a bubble-matrix chart (Fig. 2A). Clear differences between AmyM and XynA overexpression are the induction of the CssR controlled secretion stress response and YuxN regulon upon AmyM overproduction and the activation of the AscR sulphur metabolism regulon, ImmR and SknR regulon genes upon XynA overproduction. AscR activates the alkyl-sulphur catabolism *snaA* operon, which is induced by methionine but suppressed by sulfate [24, 25]. The ImmR and SknR regulon genes are part of the mobile genetic element ICEBs1 and skin element, respectively [26]. YuxN is the repressor of *yirB* [26], and activation of *yirB* expression reduces the stability of SpX [27], a global regulator of genes involved in the prevention of protein aggregation during severe heat stress and protection against paraquat stress. Importantly, the CtsR regulon genes are downregulated under all conditions. To test whether inactivation of CtsR increases AmyM production, like it did in case of XynA [10], we deleted *ctsR* in the AmyM overexpressing strain. However, this mutation gave no improvement and even resulted in a slightly lower AmyM activity (Fig. 3).

### Upregulation of other protein chaperone genes

**Fig. 4:**
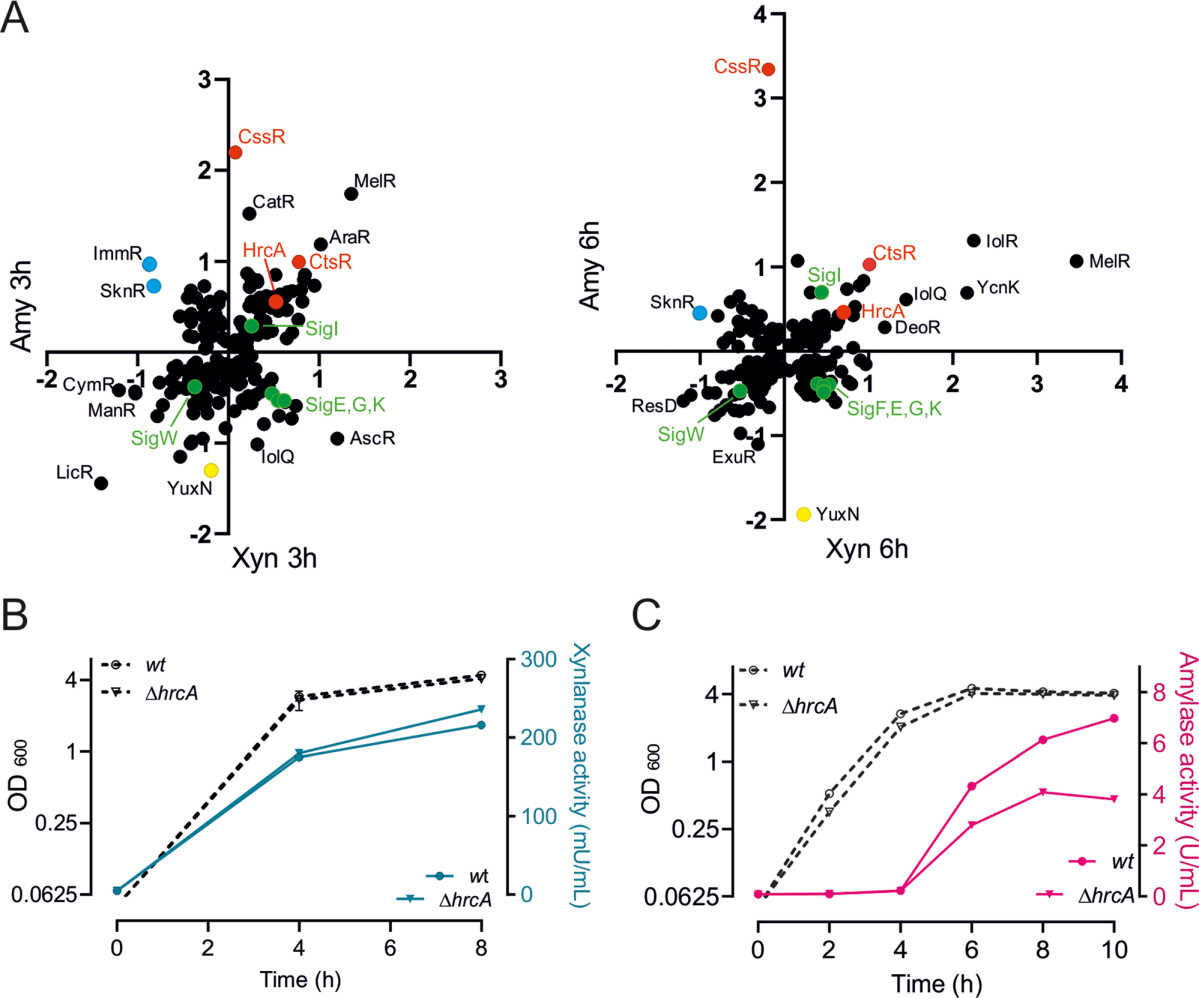
Effect of *hrcA* inactivation on XynA and AmyM production. (**A**) Scatter plot comparison of average fold changes of regulons between XynA and AmyM overexpressing cells after 3 h and 6 h growth. The regulation directionality of regulators was taken into account when calculating the average regulon activities. Relevant regulons are highlighted by different colours. (**B**) Growth (OD_600_) and xylanase activity in the medium of strains BWB09/pCS58 (*wt*) and SGB04/pCS58 (∆*hrcA*) overexpressing XynA. (**C**) Growth (OD_600_) and amylase activity in the medium of strains BWB09/pCS74 (*wt*) and SGB04/pCS74 (∆*hrcA*) overexpressing AmyM. Data shown as mean ± standard deviation of two biological replicates

To compare the effects on all regulon activities between conditions, we plotted the average fold change of AmyM overexpressing cells against that of XynA overexpressing cells in scatter plots (Fig. 4A). Interestingly, it appeared that both when AmyM and XynA were overproduced, another protein chaperone repressor, HrcA, was also slightly activated (Fig. 4A). HrcA controls expression of the conserved protein chaperones GroEL/ES as well as DnaJ and DnaK [13, 22]. The increased HrcA activity is expected to lead to a downregulation of these crucial protein chaperone genes. To examine whether overexpression of these chaperone genes, by inactivating HrcA could enhance the production of AmyM or XynA, we introduced the overexpression plasmids in a strain with a deletion of *hrcA*. In this strain XynA levels increased barely, by approximately 8% (Fig. 4B), whereas the AmyM activity dropped considerably, by about 45% (Fig. 4C).

### Effect of feeding proteases

**Fig. 5:**
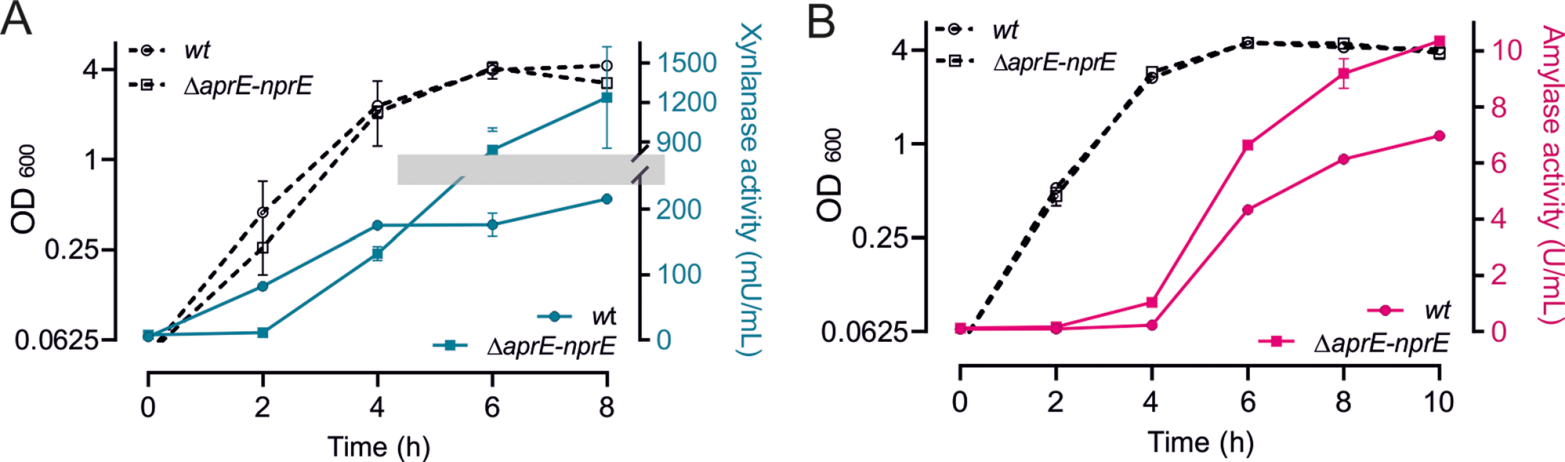
Effect of feeding proteases inactivation on XynA and AmyM production. (**A**) Growth (OD_600_) and xylanase activity of *wt* (BWB09/pCS58) and ∆*aprE* ∆*nprE* protease mutant (BWB143/pCS58). Note the interrupted line in the enzyme activity of the mutant, which is caused by the interrupted Y-axis scale (grey bar). (**B**) Growth (OD_600_) and amylase activity of *wt* (BWB09/pCS73) and ∆*aprE* ∆*nprE* protease mutant (BWB143/pCS73). Data shown as mean ± standard deviation of two biological replicates

Generally, the secretion of degradative enzymes by *Bacillus* species commences when optimal growth ceases and cells enter stationary growth. A good example is the secretion of amylases like AmyM. In contrast, the production of XynA goes down at the stationary growth phase, as shown in Fig. 1. Interestingly, this is not due to transcriptional regulation since both genes are expressed from the same promoter and mRNA levels are still high after 6 h growth (Table 2). A likely explanation is that XynA is sensitive to proteases secreted during stationary growth. These proteases are induced when nutrient becomes limiting to release peptides from proteinaceous food sources, and they are also referred to as feeding proteases [28–30]. Indeed, when we used a strain lacking the main extracellular feeding proteases NprE and AprE [28], the production of xylanase increased more than 5 fold after 8 h of growth (Fig. 5A). For AmyM, this effect was more modest and the amylase activity after 8 h of growth increased by approximately 50% (Fig. 5B).

### Ribosome profiling of *xynA*

**Fig. 6:**
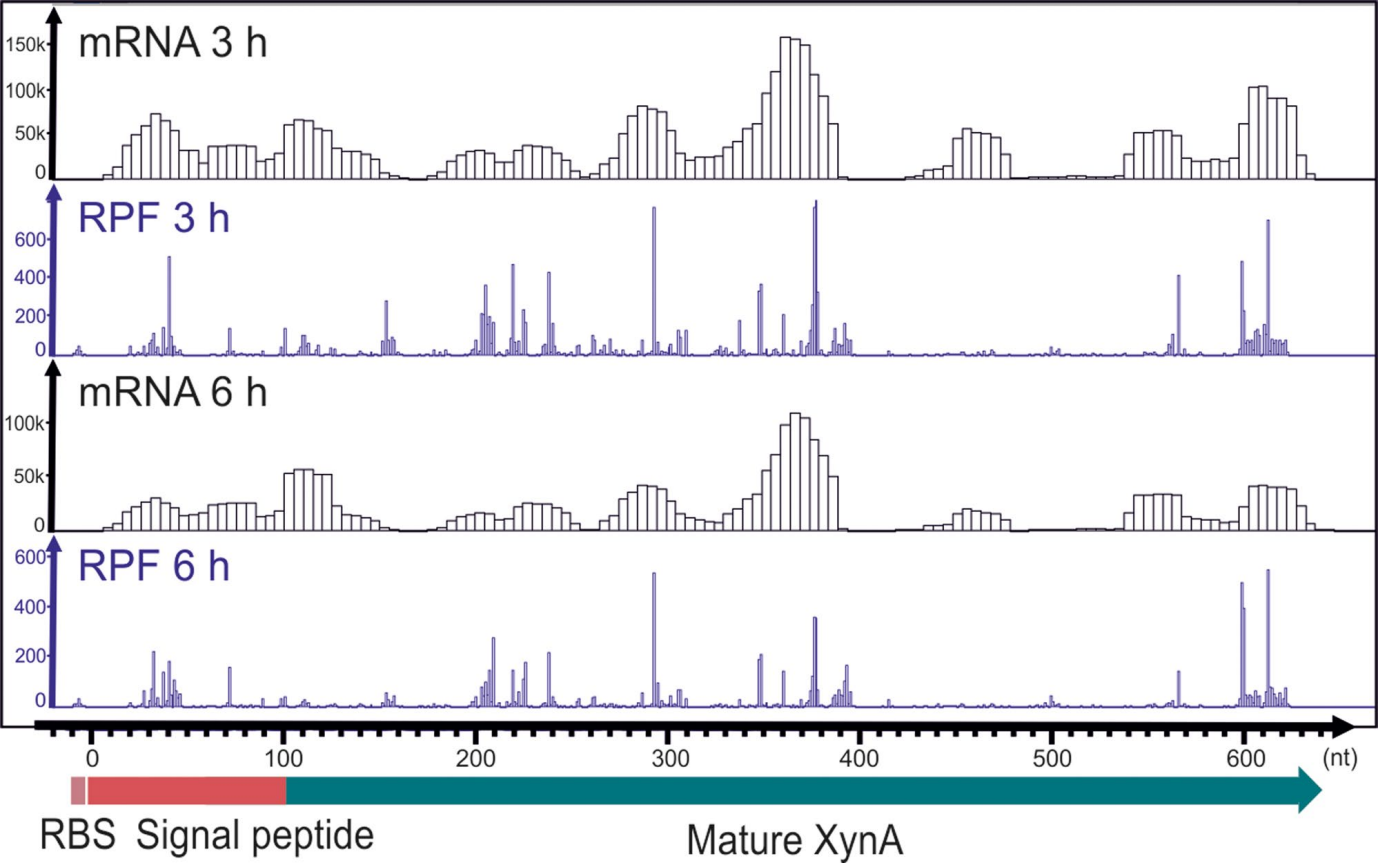
Transcriptome (mRNA) and ribosome (RPF) profiles of *xynA* at 3 h and 6 h growth. Samples were taken from the ∆*aprE* ∆*nprE* protease mutant strain BWB143 containing XynA overproduction plasmid pCS58. The profiles represent the mean of normalized reads mapped to the *xynA* locus from two independent replicates. Transcriptome (mRNA) and ribosome (RPF) profiles of *xynA* for each independent replicate samples are provided in Figure S4

Despite the observed increase in xylanase activity in the double protease mutant, the increase in XynA production between 4 h and 6 h is much higher than between 6 h and 8 h growth (Fig. 5A). This raised the question whether translation of *xynA* mRNA is reduced late in stationary phase. To investigate this, we conducted a ribosome profiling analysis [16, 31]. Total RNA and ribosomes were isolated at 3 h and 6 h growth, and a PCA analysis showed good biological replicate data (Figure S3). The normalized ribosome protected fragment reads were plotted onto the *xynA* mRNA to reveal the translation activity difference between 3 h and 6 h growth (Fig. 6). Clearly, the translation activity of *xynA* is largely the same in both samples, indicating that the limited increase in xylanase after 6 h cannot be explained by lower translation activities.

### Inactivation of LonA increases enzyme production

**Fig. 7:**
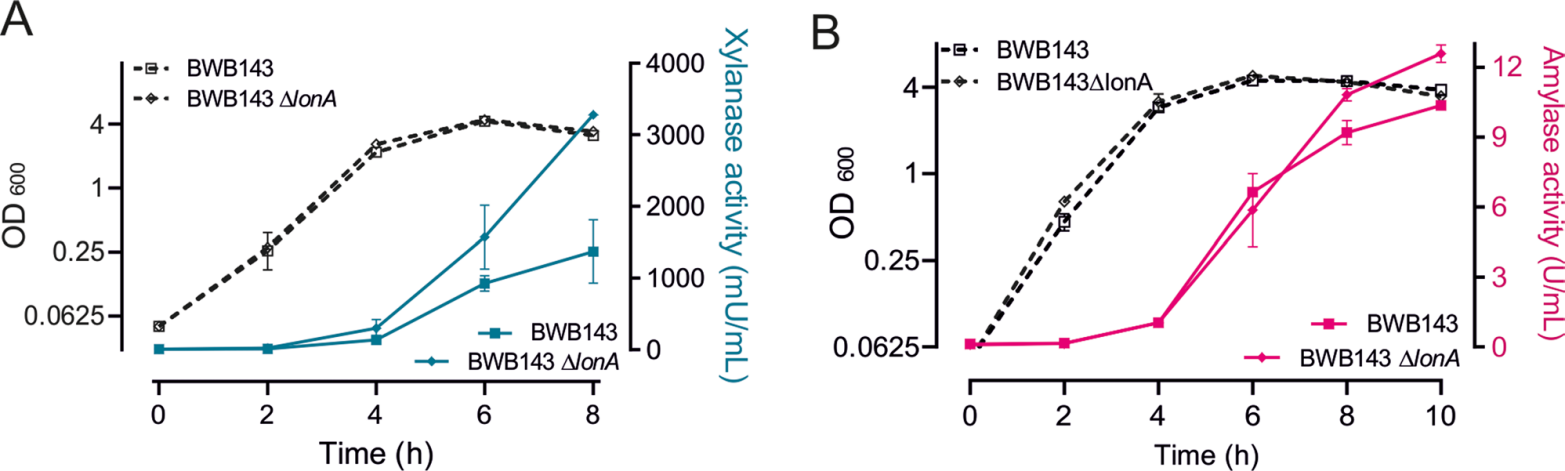
Effect of LonA inactivation on the production of XynA and AmyM. (**A**) Growth (OD_600_) and xylanase activity of ∆*aprE* ∆*nprE* protease mutant strains BWB143 with XynA overproduction plasmid pCS58, and of BWB143 ∆*lonA* strain with pCS58. (**B**) Growth (OD_600_) and amylase activity of ∆*aprE* ∆*nprE* protease mutant strains BWB143 with AmyM overproduction plasmid pCS74, and of BWB143 ∆*lonA* strain with pCS74. Data shown as mean ± standard deviation of two biological replicates

A possible explanation for the low production of XynA after 6 h could be degradation by intracellular proteases. In *E. coli* it has been shown that inactivation of the ubiquitous cytoplasmic ATP-dependent serine protease LonA can increase the expression of the *Vibrio fischeri lux* operon by reducing degradation of *Vibrio fischeri* luciferase [32, 33]. Similarly, *Thermus thermophilus* mutants deficient in Lon protease exhibit elevated production of heterologous proteins, including *Pyrococcus horikoshii* α-mannosidase and AmyM [34]. LonA of *B. subtilis* contains a classic unfoldase and protease domain [35, 36], and we wondered whether this protease might be responsible for the limited production of xylanase in the stationary phase. To test this, we deleted *lonA* in the ∆*nprE* ∆*aprE* background and measured the xylanase activity during growth. As shown in Fig. 7A, the absence of LonA resulted in a continuous accumulation of xylanase in the medium, improving the production 2.4 fold after 8 h growth, without impacting the growth rate. To examine whether this effect is specific to XynA production, we also introduced the *amyM* overexpressing plasmid in the ∆*nprE* ∆*aprE* ∆*lonA* strain. Also in this case the amylase activity in the medium increased, albeit only moderately, by approximately 20% (Fig. 7B).

## Discussion

In this study, we performed transcriptomics and ribosome profiling to investigate protein secretion bottlenecks. We found that overexpression of both XynA and AmyM increases the activity of the repressors CtsR and HrcA. Both are controlled by regulated proteolysis by the ClpCP protease complex [19, 37], and we speculate that the accumulation of nascent and partially unfolded XynA and AmyM occupies the ClpCP complex, as a consequence of which this protease complex has reduced capacity to degrade CtsR and HrcA, leading to increased cellular levels of these repressors. This effect might be enhanced by the resulting down regulation of *clp* genes. Possibly, some of the other observed regulations can be attributed to the reduced expression of Clp proteins, since many regulators are under control of regulated proteolysis by the Clp protease system, including MelR, ManR, CcpN, Fur, DegU, SinR and YcnK [19]. The downregulation of the SigW regulon might also be caused by this, since the anti-sigW factor RsiW is also controlled by ClpXP degradation [38].

*B. subtilis* produces several extracellular proteases when cells enter the stationary phase of growth. These feeding proteases generally interfere with production yields, and deleting the corresponding genes is the first step towards constructing an industrial relevant production strain [39, 40]. However, further genetic alterations to improve *B. subtilis* as production host has been challenging, and there are only a few reports describing genetic changes that lead to further increases in enzyme levels. Overproduction of the extracellular post-translocation molecular chaperone PrsA has been shown to improve α-amylase levels by 160% up to 500% [41, 42]. Increasing the negative charge of the cell wall, by blocking D-alanylation of cell wall teichoic acids, can improve the production of several enzymes by 37–85%, including cyclodextrin glycosyltransferase, nattokinase, α-amylase and β-mannanase [43, 44]. Increasing the negative charge of the membrane, by increasing the anionic phospholipid content, can increase the levels of α-amylase by up to 47% [45], and finally, increasing Clp protein chaperone levels in the cell has been shown to raise the production of β-xylanase by 25% [10]. Our study adds the inactivation of LonA to this list of beneficial modifications of *B. subtilis*.

The ATP-dependent protease LonA is conserved and present in most bacteria [46–48]. However, not much is known about its function in *B. subtilis*, other than that the protein has been linked to hyper-flagellation and regulation of the fore-spore specific sigma factor SigG [49, 50]. Thus far there has not been a report on improved protein production in Bacillus upon LonA inactivation. In *E. coli* LonA is involved in the degradation of mis- or unfolded proteins, including substrates of the secretion chaperone SecB, but also Ffh, the protein component of Signal Recognition Particle (SRP) [51–53]. In *Vibrio Cholerae*, LonA degrades the chaperones GroEL, DnaJ, DnaK, GrpE and the preprotein translocase subunit SecA [54]. The broad spectrum of substrates raises the possibility that the positive effect of LonA inactivation is not directly related to the degradation of AmyM and XynA, although this seems to be the most likely explanation for the observed improved secretion. In conclusion, the conserved nature of LonA makes it an attractive target for informed strain optimization when increased protein production is the aim, and in case of *Bacillus* production strains, LonA can be added to the list of proteases whose inactivation improves enzyme production.

## Materials and methods

### Bacterial strains growth conditions and mutant construction

Bacterial strains and plasmids are listed in Supplementary Table S1 and Table S2. Nutrient Luria-Bertani medium (LB, containing 10 g/L Tryptone, 5 g/L Yeast Extract, 10 g/L NaCl) was used for general growth of *B. subtilis.* Supplements were added as required: erythromycin (ery, 5 µg/mL) and kanamycin (kan, 50 µg/mL). XynA and AmyM were overexpressed in *B. subtilis* strain BWB09 using plasmid pCS58 and pCS74, respectively. The empty plasmid pBW17 was used as control. For XynA and AmyM secretion profile measurements, 10 mL overnight culture were grown in LB liquid medium supplemented with 50 µg/mL kan in 100 mL flasks at 30 °C and 210 rpm to prevent sporulation; the next morning, 1mL overnight was were quickly spin-down and the supernatant was removed and cell pellet was resuspend in 37 °C pre-warmed LB and diluted into fresh and prewarmed LB liquid supplemented with 50 µg/mL kan to a start OD600 of 0.05, grown with 210 rpm shaking at 37 °C and sampled at desired timepoint for follow-up enzymatic or protein experiments. We used 100mL flask for 10mL liquid culture and 250 mL flask for 25 mL culture to guarantee aerobic growth.

For *B. subtilis* DNA transformation, the Spizizen-plus and Spizizen-starvation media (SMM, containing 15 mM (NH_4_)_2_SO_4_, 80 mM K_2_HPO_4_, 44 mM KH_2_PO_4_, 3 mM tri-sodium citrate, 0.5% glucose, 6 mM MgSO_4_, 0.2 mg/mL tryptophan, 0.02% casamino acids, and 0.000 11% ferric ammonium citrate (NH_4_)_5_Fe(C_6_H_4_O_7_)_2_) were used and then the transformants were selected in LB-agar plate with antibiotic selection [55].

The single mutant strains ∆*hrcA* and ∆*lonA* were constructed by transformation of the chromosomal DNA from respective BKE library mutants [56] into competent BWB09 or BWB143 cells, selected via LB + Em agar plates and verified by PCR and sequencing the PCR products.

### RNA extraction

RNA extraction was based on the methods described in [57, 58]. Briefly, 2 mL cells were collected from either the logarithmic growth phase (3 h) and stationary growth phase (6 h). Cell pellets were resuspended in 0.4 mL ice-cold growth medium and added to a screw cap tube containing 1.5 g glass beads (0.1 mm), 0.4 mL phenol/chloroform/isoamyl alcohol mixture (25:24:1, Carlroth) and 50 µl 10% SDS, vortexed to mix, and stored by flash freezing in liquid nitrogen. Cell disruption was achieved by bead beating (Precellys 24). After centrifugation, RNA in the upper aqueous phase was ethanol-precipitated, washed twice with 70% ice cold ethanol, dried and dissolved in water. DNA was removed by DNAseI (NEB) treatment. The RNA was then extracted by a second-round of P/C/I extraction, followed by ethanol precipitation and 70% ethanol washing, and dissolving in water.

### RNA-seq and sequencing data analysis

Prior to the deep-sequencing, the RNA samples were treated with the MICROBExpress™ Bacterial mRNA Enrichment Kit (Thermo Fisher) to remove most of the 16 S and 23 S rRNA. Subsequently, the RNA-seq libraries were constructed using the NEBNext^®^ Ultra™ II Directional RNA Library Prep Kit from Illumina^®^ (New England Biolabs) using NEBNext^®^ Multiplex Oligos for Illumina^®^ (New England Biolabs), according to the manufacturer’s protocol. Sequencing was performed on an Illumina NextSeg 550 System using NextSeq 500/550 High Output v2.5 kit (75-bp read length), and the raw data were processed using the web-based platform Galaxy (https://usegalaxy.org/). We aimed at a sequencing depth of 5–10 million reads/library [28]. *Trimmomatic* was used to trim the adaptor sequence and filter bad reads. The trimmed reads were aligned to the Bacillus reference genome (NC_000913) with *Bowtie2*. After mapping, aligned reads were counted by *FeatureCount*, referred to the BSU locus_tags. DESeq2 was used to determine differentially expressed features between samples. A customized Excel plugin, GINtool [23], was used to analyse the transcriptome data using prior knowledge on operons, functional categories and regulons.

### Ribosome profiling

Ribosome profiling was based on the methods described in [16, 31] and a workflow diagram can be found in Figure S4. Briefly, 100 mL cells at the logarithmic growth phase (3 h) or stationary growth phase (6 h) were mixed with 0.4 mL 250 mM chloramphenicol (final concentration 1 mM) and 100 mL crushed ice made of 1mM chloramphenicol in PBS pH 7.4 and centrifuged for 5 min at 9000 xg at 4 °C. Cell pellet was subjected to flash freeze in liquid nitrogen and stored at -80 °C. The pellet was resuspended in 2 mL lysis buffer (100 mM NH_4_Cl, 10 mM MgCl_2_, 20 mM Tris pH 8.0, 0.4% Triton X-100, 0.1% NP-40, 5 mM CaCl_2_, 1 mM chloramphenicol) and pulverized cryogenically in a 25 mL stainless steel grinding jar with a 12 mm ball (Retsch) using the mixer mill MM400 (Retsch). The pulverization was achieved by 8 cycles of 2 min milling at a frequency of 20 1/s and 1 min cooling in liquid nitrogen between the cycles. The pulverized cells were thawed on ice and added with 50 µL DNase I (NEB) and 10 mL lysis buffer. The lysate was centrifuged for 10 min at 15,000 xg at 4 °C. 1 mL of the clarified supernatant was used for total RNA isolation and 9 mL of the clarified supernatant was subjected to ultracentrifugation over a 8 mL sucrose cushion (20% sucrose, 100 mM NH_4_Cl, 10 mM MgCl_2_, 20 mM Tris pH 8.0, 0.5 mM EDTA, 0.4% Triton X-100, 0.1% NP-40, 1 mM chloramphenicol) to collect ribosome pellets. The ultracentrifugation was performed using OptiSeal polypropylene tubes (Beckman) in a Ti-60 rotor at 50,000 rpm and 4 °C for 2 h. Ribosome pellets were resuspended in 200 µL resuspension buffer (100 mM NH_4_Cl, 10 mM MgCl_2_, 20 mM Tris pH 8.0) and the RNA concentration was measured using Nanodrop (Thermo Scientific). To generate monosomes from polysomes, 1 mg of ribosome RNA was digested with 8 µl micrococcal nuclease (NEB, 2000 U/ µL) at 37 °C and after 30 min 8 µL 0.25 M EGTA was added to quench the reaction. The digested sample was subjected to ultracentrifugation over a sucrose gradient solution (10 − 50% sucrose in 100 mM NH_4_Cl, 10 mM MgCl_2_, 20 mM Tris pH 8.0, 2 mM DTT) to isolate monosomes from polysomes. The ultracentrifugation was performed using Open-Top thin-wall polypropylene tubes (Beckman) with 0.9 mL of 10–50% sucrose solutions (4.5 mL in total) from the bottom in an SWTi-55 rotor at 42,000 rpm and 4 °C for 2.5 h. The 0.9 mL of the 30%, 40% and 50% sucrose fractions were collected and subjected to RNA extraction using P/C/I (Carlroth), followed by isopropanol precipitation and 70% ethanol washing, and resuspended in 8 µL nuclease free water. The RNA sample was separated by electrophoresis on a 15% TBE 7 M Urea PAGE gel at 60 V for 20 min and then at 180 V for 1 h. The ribosome protected fragments (RPFs) of size between 22 nt and 34 nt were excised from the gel, purified and subjected to sequencing library construction using NEBNext^®^ Small RNA Library Prep Set for Illumina (NEB) according to the manufacturer’s instructions.

The total RNA from clarified lysate supernatant was purified using P/C/I method and the mRNA was enriched via the RNaseH-mediated rRNA depletion. The enriched mRNA was fragmented using Magnesium RNA Fragmentation Module (NEB) and subjected to electrophoresis separation. Similarly, the fragmented mRNAs of size between 22 nt and 34 nt were excised from the gel, purified and subjected to sequencing library construction, as vehicle control. Sequencing was also performed on an Illumina NextSeg 550 System using NextSeq 500/550 High Output v2.5 kit.

### Ribosome profiling data analysis

The raw sequencing data were processed using the Galaxy platform and the RiboGalaxy platform (https://ribogalaxy.ucc.ie/). On the Galaxy platform, *Cutadapt* was used to remove 3’ adapter sequences and select reads of size between 18 and 32 nucleotide [59]. *FastQC* was used to check the read quality [60], the *Bowtie2* was used to align reads to the *B. subtilis* genome sequence NC_000964.3 to generate SAM files [61], which link reads to their genomic position. The SAM files were then converted to BAM format using *Filter SAM or BAM* (MAPQ > = 3) [62]. The aligned reads were counted by *FeatureCount* [63]. We processed the BAM files on the RiboGalaxy platform for further analysis.

### Xylanase and amylase enzyme activity assays

100 µl cells were taken from the culture transferred into a 1.5 mL Eppendorf tube and centrifuged at 20,000 RCF for 1 min at 4 °C, and then 70 µl supernatant was transferred to a new tube and stored by flash freezing in liquid nitrogen and storage at -80 °C. Xylanase enzyme activity in the supernatant was determined using the fluorescence-based assay EnzChek^®^ Ultra Xylanase Assay Kit (Thermo Fisher Scientific), according to the manufacturer’s instructions. Amylase enzyme activity were tested using α-Amylase Assay Kit (Megazyme, Ceralpha Method #K-CERA) in a optimized and scaled-down system based on the manufacturer’s instructions. Briefly, 50 µL substrate was reacted with 50 µL AmyM samples at 25 °C for 20 min in a 96-well plate, and then 100 µL Tris-Stop solution (20 g/L Tris in pH 10.0) was added to stop the reaction. The absorbance (405 nm) was measured using a Multiskan FC microplate photometer (Thermo Fischer Scientific, #51119000). All supernatant samples were only thawed on ice-water prior to the test. The commercial Xylanase (Sigma, X2753) and α-Amylase from *Bacillus subtilis* (Sigma, 10069) were used for construction of standard curve for the detection of xylanase and amylase enzyme activity respectively.

## Electronic supplementary material

Below is the link to the electronic supplementary material.

Supplementary Material 1

## Data Availability

RNA-seq and Ribosome profiling data have been submitted to and are accessible in the Gene Expression Omnibus (GEO), accession number GSE270692.
