# Supplementary material for: Inactivation of the conserved protease LonA increases production of xylanase and amylase in Bacillus subtilis
Source: Microb Cell Fact. 2024 Dec 19;23:335. doi: 10.1186/s12934-024-02616-6 (PMC11656851; doi:10.1186/s12934-024-02616-6)
Supplement: Supplementary file 1 — Supplementary Material 1 [file 12934_2024_2616_MOESM1_ESM.docx]

**Inactivation of the conserved protease LonA increases production of xylanase and amylase in *Bacillus subtilis***

Biwen Wang, Mariah B.M.J. Kes, Anna C.H. van den Berg van Saparoea, Gaurav Dugar, Joen Luirink, Leendert Hamoen

**Content**

- Table S1. Strains used in this study
- Table S2. Plasmids used in this study
- Figure S1. Principal component analysis (PCA) of RNA-seq samples
- Figure S2. Workflow of Ribo-seq
- Figure S3. Principal component analysis (PCA) of ribosome profiling samples
- Figure S4. Transcriptome (mRNA) and ribosome (RPF) profiles of *xynA* from two independent replicate samples at 3 h and 6 h growth.
- Supplementary references

**Table S1.** Strains used in this study

| **Strain** | **Genotype** | **Source or reference** |
| --- | --- | --- |
| BSB1 | *B. subtilis* wildtype strain, *trp+* | Lab strain |
| BWB09 | BSB1 ∆*xynA* ∆*amyE* | [1] |
| SGB03 | BSB1 ∆*xynA* ∆*amyE ∆ctsR::ery* | [1] |
| SGB04 | BSB1 ∆*xynA* ∆*amyE ∆hrcA::ery* | This study |
| BWB143 | BSB1 ∆*nprE* ∆*aprE* ∆*spoIIE* | [2] |
| BWB145 | BSB1 ∆*nprE* ∆*aprE* ∆*spoIIE ∆lonA::ery* | This study |
| BKE-168 | *B. subtilis* 168 strain (BGSC, 1A1) | BGSC[3,4] |
| BKE28200 | 1A1, ∆*lonA*::ery | BGSC[3,4] |

**Table S2.** Plasmids used in this study.

| **Plasmid** | **Genotype** | **Source/ reference** |
| --- | --- | --- |
| pBW17 | Empty control plasmid with P*_amyQ_* promoter, start and stop codon | This study |
| pCS58 | Plasmid overexpressing XynA with P*_amyQ_* promoter | [1] |
| pCS74 | Plasmid overexpressing AmyM with P*_amyQ_* promoter | [5] |

**Figure S1.**


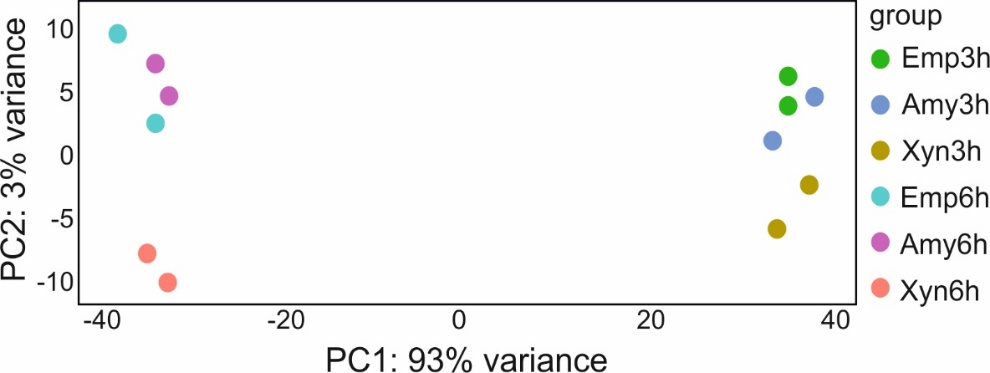


**Figure S1.** Principal component analysis of RNA-seq samples. RNA samples were extracted from BWB09/pCS74 (Amy), BWB09/pCS58 (Xyn) and control BWB09/pBW17 (Emp) cells at 3 h and 6 h growth, respectively. Each sample group at each time point contains 2 independent biological replicates. Data of the 3 h XynA sample were obtained from [1].

**Figure S2.**


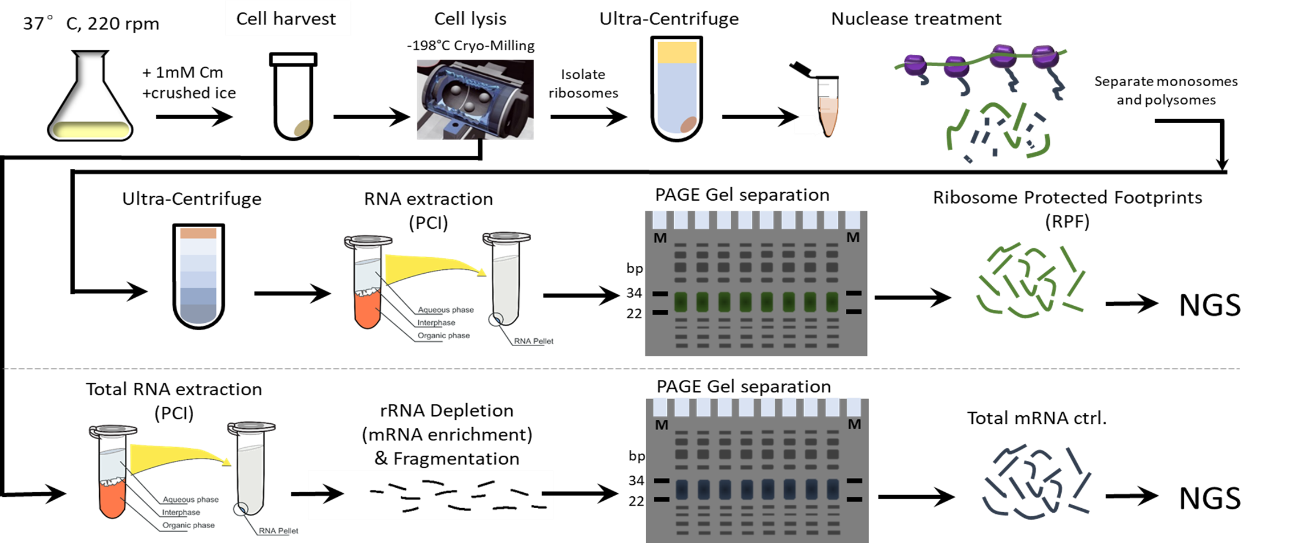


**Figure S2.** Workflow of ribosome profiling. Briefly, 100 ml cells were mixed with 0.4 mL 250 mM chloramphenicol and 100 mL crushed ice. Cell pellet was subjected to flash freezing in liquid nitrogen and stored at -80 °C. The pellet was resuspended and cryogenically pulverized. The pulverized cells were subject to ribosome protected fragment (RPF) purification or total RNA extraction. For RPF purification, clarified supernatants were obtained and subjected to ultracentrifugation over a sucrose cushion (20 % sucrose to collect ribosome pellets). Ribosome pellets were digested with micrococcal nuclease to generate RPFs. The digested samples were ultracentrifuged over a sucrose gradient solution to isolate monosomes. RNA was extracted from monosomes and then separated by electrophoresis. The RPFs of size between 22 nt and 34 nt were excised and purified from the gel, and subjected to sequencing library construction. For total RNA extraction, the RNA was purified from clarified lysate supernatant using P/C/I and the mRNA was enriched via RNaseH-mediated rRNA depletion. Enriched mRNA was subsequently fragmented using Magnesium RNA Fragmentation Module (NEB) and subjected to electrophoresis separation, and sizes between 22 nt and 34 nt were excised and purified from the gel, and subjected to sequencing library construction.

**Figure S3.**


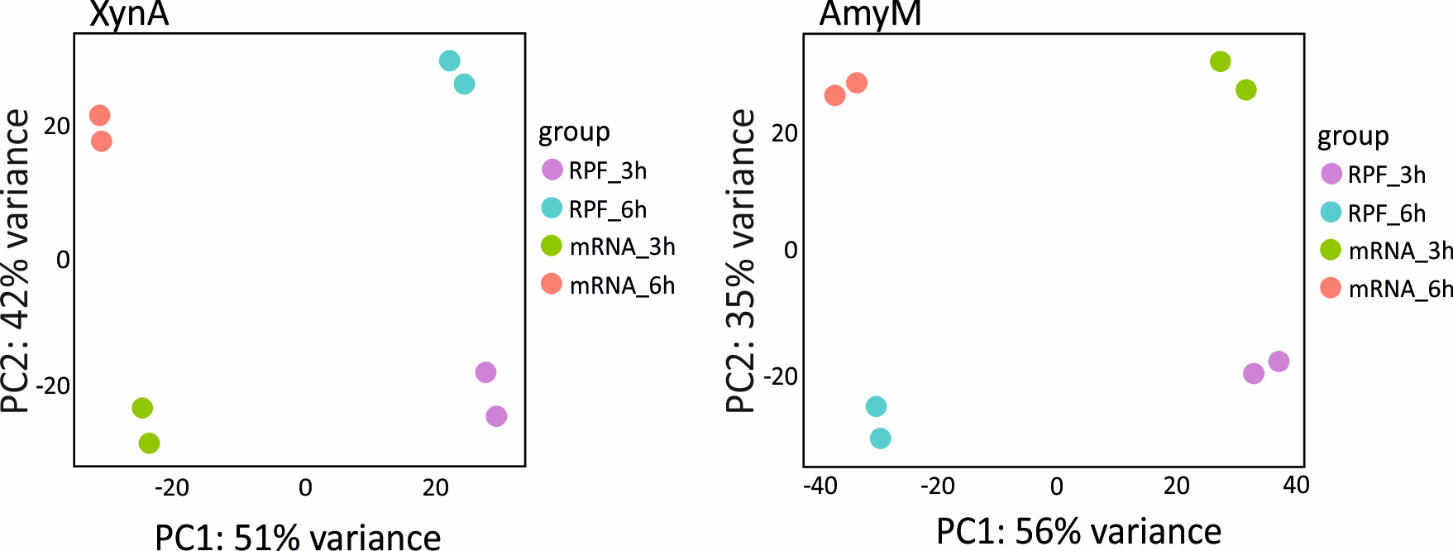


**Figure S3.** Principal component analysis (PCA) of ribosome profiling samples. Ribosome protected fragments and mRNA samples were extracted from BWB143/pCS58 (XynA) cells at 3 h and 6 h growth, respectively. Each sample group at each time point contains 2 independent biological replicates.

**Figure S4.**


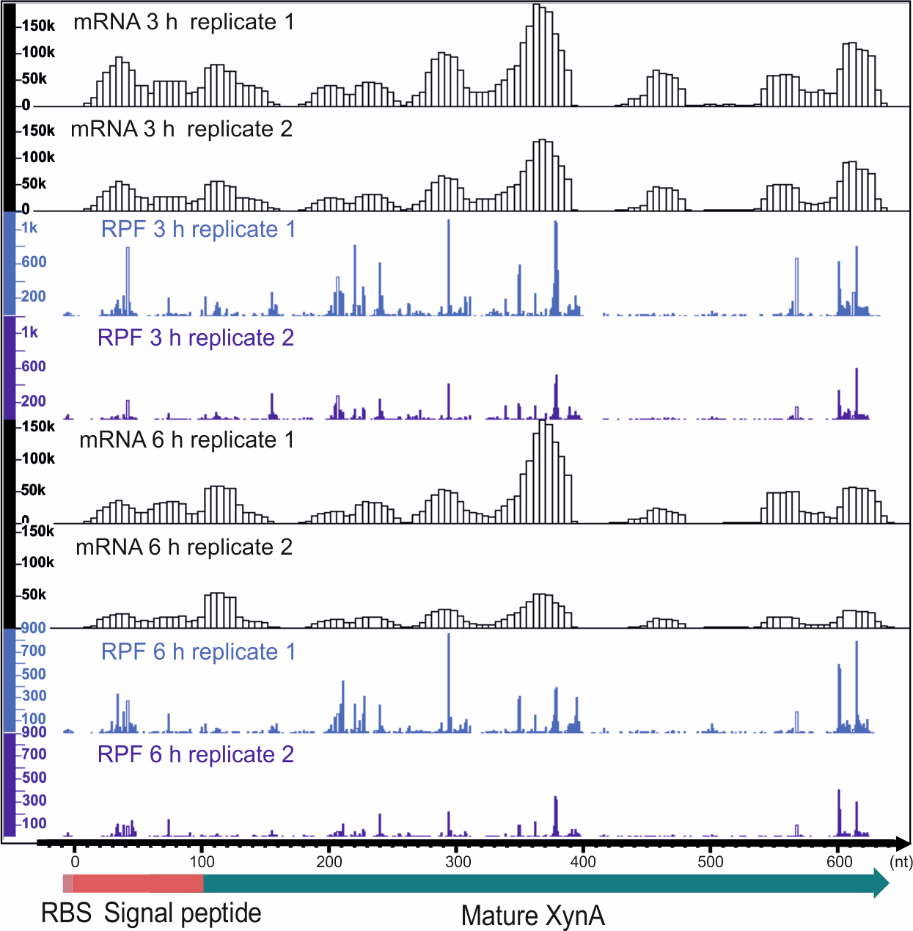


**Figure S4.** Transcriptome (mRNA) and ribosome (RPF) profiles of *xynA* from two independent replicate samples at 3 h and 6 h growth. Data shown are normalized reads mapped to the *xynA* locus.

**Supplementary references**

1. Wang B, van der Kloet F, Hamoen LW. Induction of the CtsR regulon improves Xylanase production in *Bacillus subtilis*. Microbial Cell Factories. 2023;22:231.

2. Kes MBMJ, Wang B, van Ulsen P, Hamoen LW, Luirink J. Development of a split-luciferase assay to establish optimal protein secretion conditions for protein production by *Bacillus subtilis*. Microbiology. 2024;170:001460.

3. Kunst F, Ogasawara N, Moszer I, Albertini AM, Alloni G, Azevedo V, et al. The complete genome sequence of the Gram-positive bacterium *Bacillus subtilis*. Nature. 1997;390:249–56.

4. Koo B-M, Kritikos G, Farelli JD, Todor H, Tong K, Kimsey H, et al. Construction and analysis of two genome-scale deletion libraries for *Bacillus subtilis*. Cell systems. 2017;4:291–305.

5. Henriques G, McGovern S, Neef J, Antelo-Varela M, Götz F, Otto A, et al. SppI Forms a Membrane Protein Complex with SppA and Inhibits Its Protease Activity in *Bacillus subtilis.* mSphere. 2020;5:10.1128/msphere.00724-20.
